# IL-17 and IFN-γ–producing Respiratory Tissue-Resident Memory CD4 T Cells Persist for Decades in Adults Immunized as Children With Whole-Cell Pertussis Vaccines

**DOI:** 10.1093/infdis/jiae034

**Published:** 2024-01-30

**Authors:** Karen N McCarthy, Stephen Hone, Rachel M McLoughlin, Kingston H G Mills

**Affiliations:** School of Biochemistry and Immunology, Trinity Biomedical Sciences Institute, Trinity College Dublin, Dublin, Ireland; Children's Health Ireland, Dublin, Ireland; Children's Health Ireland, Dublin, Ireland; Department of Otolaryngology, Royal Victoria Eye and Ear Hospital, Dublin, Ireland; School of Medicine, University College Dublin, Dublin, Ireland; School of Biochemistry and Immunology, Trinity Biomedical Sciences Institute, Trinity College Dublin, Dublin, Ireland; School of Biochemistry and Immunology, Trinity Biomedical Sciences Institute, Trinity College Dublin, Dublin, Ireland

**Keywords:** *Bordetella pertussis*, whole-cell pertussis vaccine, acellular pertussis vaccine, tissue-resident memory T cell, mucosal immunity, Th1 cell, Th17 cell

## Abstract

The objective was to determine if antigen-specific tissue-resident memory T (T_RM_) cells persist in respiratory tissues of adults immunized as children with whole-cell pertussis (wP) or acellular pertussis (aP) vaccines. Mononuclear cells from tonsil or nasal tissue cells were cultured with *Bordetella pertussis* antigens and T_RM_ cells quantified by flow cytometry. Adults immunized with wP vaccines as children had significantly more interleukin 17A (IL-17A) and interferon-γ (IFN-γ)–producing T_RM_ cells that respond to *B. pertussis* antigens in respiratory tissues when compared with aP-primed donors. Our findings demonstrate that wP vaccines induce CD4 T_RM_ cells that can persist in respiratory tissues for decades.

## Teaser

Most studies on T-cell responses to mucosal pathogens focus on the blood. Here we demonstrate tissue-resident memory T cells persist in the tonsil and nasal tissue of humans for decades after immunization with whole-cell pertussis vaccines.

## Graphical Abstract

**Graphical Abstract:**
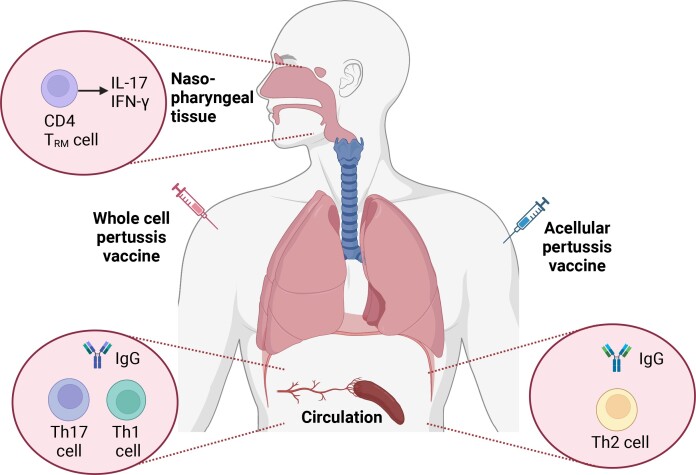

*Bordetella pertussis* infects the human respiratory tract causing whooping cough, a disease most severe in infants and unvaccinated children. Whole-cell pertussis (wP) vaccines, introduced in the 1950s, are highly effective in reducing disease burden; however, concerns about adverse events led to the development of safer acellular pertussis (aP) vaccines. Despite excellent global vaccine coverage, pertussis continues to contribute a significant disease burden [1]. aP vaccines prevent pertussis disease and mortality, but do not prevent asymptomatic carriage and this may be driving the increased incidence among vulnerable infants [2, 3]. Studies in animal models have demonstrated that Th1 and Th17 cells induced following infection or immunization with wP vaccines confer protective immunity against *B*. *pertussis* [4, 5]. In contrast, aP vaccines induce Th2-polarized systemic immune responses in mice and humans and this, together with waning immunity, may explain the poorer efficacy of these vaccines [6, 7].

Respiratory tissue-resident memory T cells (T_RM_) have emerged as a pivotal lymphocyte population in long-term immunity to *B. pertussis*. Studies in mice have demonstrated that natural infection with *B. pertussis* induces antigen-specific interleukin 17 (IL-17)– and/or interferon-γ (IFN-γ)–secreting CD4 T_RM_ that persist in the lung and nasal cavity, and subsequently proliferate locally and mediate rapid clearance of bacteria following reinfection [4, 8, 9]. Immunization of mice with wP vaccines also prime IFN-γ and IL-17–secreting respiratory T_RM_ cells, that expand locally following infection with *B. pertussis* and promote bacterial clearance from lungs and nasal mucosa [4]. However, T_RM_ cells are not generated following immunization with aP vaccines, and there is evidence that infection-induced T_RM_ cells are suppressed; this may explain the failure of these vaccines to prevent nasal infection with *B. pertussis* [4, 10].

The objective of this study was to examine if antigen-specific T_RM_ cells were detectable in respiratory tissue of adults immunized with pertussis vaccines as children and to determine the cytokine profile of antigen-stimulated T_RM_ cells induced with wP or aP vaccines.

## METHODS

These studies were approved by the Faculty of Health Sciences Ethics Committee, Trinity College Dublin and the Clinical Research Ethics Committee, Royal Victoria Eye and Ear Hospital (RVEEH), Dublin. Informed consent was obtained from all study participants. Patients scheduled to undergo tonsillectomy for recurrent tonsillitis at RVEEH or healthy volunteers were recruited. Nasal tissue cells (NTC) were obtained from another cohort of healthy volunteers. Matched blood samples were collected from both cohorts. Patient demographics and exclusion criteria are in Supplementary Tables 1 and 2.

Tonsil tissues were mechanically disrupted using a scalpel and passed through a nylon sieve. Peripheral blood mononuclear cells (PBMC) or tonsil mononuclear cells (TMC) were isolated using density gradient centrifugation. TMC or PBMC (1 × 10^6^) were incubated with TexMax medium (negative control; Miltenyi Biotech), staphylococcal enterotoxin B (SEB; 1 µg/mL; positive control), heat-killed sonicated *B. pertussis* (SBP; 10 µg/mL), or purified filamentous hemagglutinin (FHA; 1 µg/mL). FHA induced more consistent T-cell responses than pertussis toxin (PT). Anti-CD28 and anti-CD49d (1 µg/mL) were added as cosimulants and cells incubated at 37°C for 18 hours. Brefeldin A (5 µg/mL) and Monensin (5 µg/mL) were added 4 hours prior to the end of culture, followed by surface staining for CD3, CD4, CD8, CD45R0, CCR7, CD69, and CD103. Cells were fixed and permeabilized and intracellular cytokine staining performed for IL-17A, IFN-γ, and IL-13. Details of antibodies and other reagents are provided in Supplementary Table 3.

NTC were obtained using nylon flocked midturbinate swabs [11]. Swabs were placed in tubes with TexMax (Miltenyi Biotech) medium on ice and NTC cells isolated within 1 hour by vortexing, followed by centrifugation. NTC (2 × 10^5^/mL) were cultured at 37°C for 18 hours with medium (TexMax; Miltenyi Biotech), human CytoStim (positive control; Miltenyi Biotec), or SBP and autologous antigen-presenting cells (4 × 10^5^/mL) generated by depleting CD3 T cells from matched PBMC by magnetic-activated cell sorting and irradiating at 40 Gy using a Gammacell irradiator. IFN-γ and IL-17A–secreting cells were detected by flow cytometry using cytokine secretion assays following the supplier's instructions (Miltenyi Biotech). This sensitive assay captures cytokines secreted by cells using bispecific antibodies against CD45 on the cell surface and the cytokine of interest.

TMC, NTC, and PBMC cells were acquired on a Fortessa Flow Cytometer (BD) using FACS DIVA software (BD) and analyzed on FlowJo software (Tree Star). Gating strategies are shown in Supplementary Figures 1 and 2. The frequency of antigen-specific cytokine-producing T cells was determined by subtracting responses for medium control from antigen-stimulated cells. Anti-PT serum immunoglobulin G (IgG) levels were measured using Euroimmun enzyme-linked immunosorbent assay (ELISA).

Statistical analysis was performed using GraphPad Prism (GraphPad Software). Data from 2 unpaired groups were analyzed using Mann-Whitney test. *P* < .05 was considered to be statistically significant.

## RESULTS

### *B. pertussis*-Specific CD4 T_RM_ Cells Are Present in Human Tonsil

Tonsils provide a rich source of immune cells to assess respiratory T-cell responses to *B. pertussis* in humans. Twenty adult patients (10 wP vaccinated and 10 aP vaccinated as children) undergoing elective tonsillectomy were recruited and tonsil tissue and blood collected. No patients had a positive *B. pertussis* polymerase chain reaction (PCR) from nasopharyngeal swab at the time of procedure, recent subclinical infection with *B. pertussis* based on anti-PT serum IgG (Supplementary Table 1), or a prior history of known *B. pertussis* infection.

**Figure 1.**
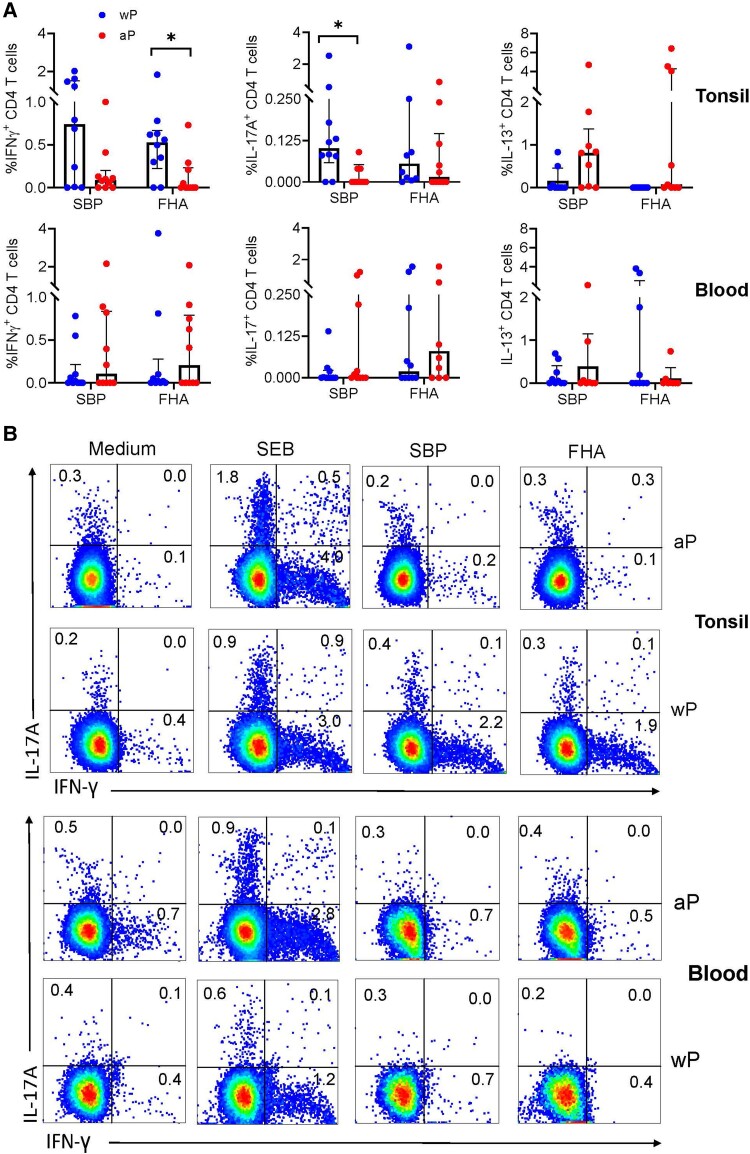
Significantly higher frequency of IFN-γ and IL-17A–producing CD4 T cells were found in tonsil of adults primed with wP compared with aP vaccines as children. Tonsil and blood samples were isolated from adult donors who had received wP (n = 10) or aP (n = 10) vaccines as children. Peripheral blood mononuclear cells or tonsil mononuclear cells were stimulated with medium (negative control), SEB (positive control), SBP, or FHA, and cytokine-producing CD4 T cells were quantified by surface staining, intracellular cytokine staining, and flow cytometry. *A*, Frequency of IFN-γ^+^, IL-17A^+^, or IL-13^+^ CD4 T cells that respond to SBP or FHA in blood and tonsil (the frequency of cytokine-positive cells in the medium control was subtracted from the frequency for antigen-stimulated cells). *B*, Sample flow cytometry plots. Data shown are median and interquartile range. *P < .05, Mann-Whitney test. Abbreviations: aP, acellular pertussis; FHA, filamentous hemagglutinin; IFN-γ, interferon-γ; IL, interleukin; SBP, sonicated *Bordetella pertussis*; SEB, staphylococcal enterotoxin B; wP, whole-cell pertussis.

We first compared total CD4 T cells responding to *B. pertussis* antigens in tonsil and blood. CD4 T cells in a high number of tonsil samples produced IFN-γ and IL-17 following in vitro stimulation with SBP or FHA. The frequency of IL-17^+^ CD4 T cells was significantly higher in donors immunized with wP compared with aP vaccines (Figure 1*A* and 1*B*). The frequency of CD4 T cells producing IFN-γ in response to FHA was also significantly higher in donors immunized with wP compared with aP vaccines. In contrast, IL-13–producing CD4 T cells responding to SBP or FHA were significantly higher from aP-vaccinated donors. Antigen-specific CD4 T-cell responses were detectable in a smaller proportion of PBMC samples, and there were no significant differences in IL-17, IFN-γ, or IL-13 production by peripheral CD4 T cells from individuals that received wP or aP vaccines as children (Figure 1*A* and 1*B*).

**Figure 2.**
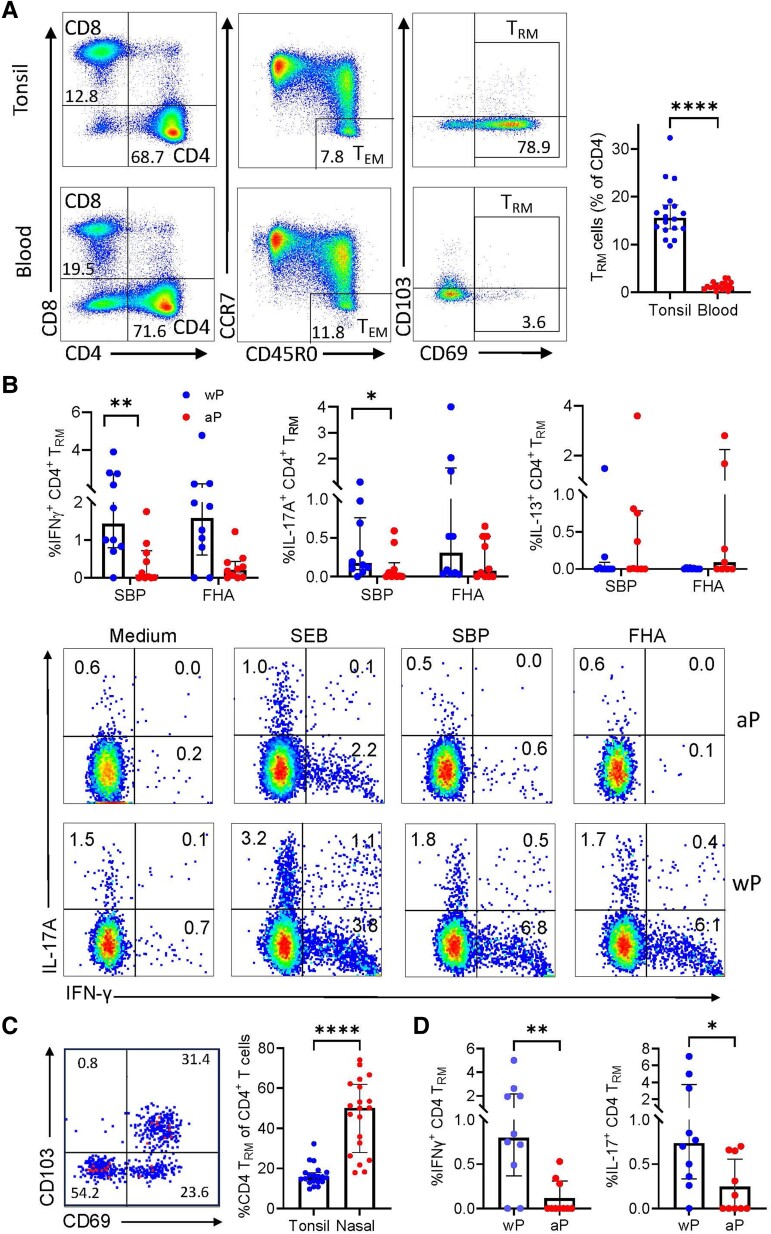
IFN-γ and IL-17A–producing CD4 T_RM_ that respond to *Bordetella pertussis* antigens in tonsil and nasal tissue of adults primed with wP but not aP vaccines as children. Tonsil and blood samples were isolated from adult donors who received wP (n = 10) or aP (n = 10) vaccines as infants. *A*, CD4^+^, CD8^+^ T cells (of total CD3^+^ cells), CD4 T_EM_ (CD45R0^+^ CCR7^−^), and CD4 T_RM_ cells (CD69^+^ CD103^+/−^) in tonsil versus blood, with median frequency of total CD4 T_RM_ cells in tonsil versus blood in histogram to right. *B*, Tonsil mononuclear cells were stimulated with medium (negative control), SEB (positive control), SBP, or FHA and cytokine-producing CD4 T_RM_ cells were quantified by surface staining, intracellular cytokine staining, and flow cytometry. Results are shown as mean frequency of IFN-γ^+^, IL-17A^+^, and IL-13^+^ antigen-specific CD4 T_RM_ cells with sample flow cytometry plots below. *C*, Nasal tissue cells were isolated by nasal swabbing from normal adult donors who received wP (n = 10) or aP (n = 10) vaccines as children. Flow cytometry plots showing CD4 T_RM_ cells (CD69^+^CD103^+/−^) in nasal tissue with histogram to right showing the frequency of total CD4 T_RM_ cells in nasal tissue compared with tonsil. *D*, NTC were stimulated with medium (negative control), CytoStim (positive control) SBP, and cytokine-producing CD4 T_RM_ cells were quantified by surface staining, cytokine capture assay, and flow cytometry. Results are mean frequency of IFN-γ^+^, IL-17A^+^ CD4 T_RM_ cells that respond to SBP. The frequency of cytokine-positive cells in the medium control was subtracted from the frequency for antigen-stimulated cells. Data shown are median and interquartile range. *P < .05, **P < .01, **** *P* < .0001, Mann-Whitney test. Abbreviations: aP, acellular pertussis; FHA, filamentous hemagglutinin; IFN-γ, interferon-γ; IL, interleukin; SBP, sonicated *B. pertussis*; SEB, staphylococcal enterotoxin B; T_Rm_, tissue-resident memory T cell; wP, whole-cell pertussis.

The frequencies of CD4 and CD8 T cells and the number of effector memory T (T_EM_) cells was similar in tonsil and blood (Figure 2*A*). In contrast, the number of CD4 T_RM_ cells, defined as CD4^+^CD45RO^+^CCR7^−^CD69^+^CD103^+/−^, was significantly higher in tonsil compared with blood (Figure 2*A*). Assessment of antigen-specific T_RM_ cells in tonsils revealed significantly higher frequencies of IFN-γ and IL-17–producing CD4 T_RM_ cells responding to SBP or FHA (Figure 2*B*) in individuals that received wP compared with aP vaccines. In contrast, there were no significant differences in IL-13^+^ CD4 T_RM_ cells between aP and wP primed donors. However, we did detect IL-13–secreting CD69^−^CD103^−^ responding to SBP in donors immunized with aP (data not shown), suggesting these were circulating T cells or tissue-resident cells that are not T_RM_ cells.

### CD4 T_RM_ Cells That Respond to *B. pertussis* Antigens Are Present in Nasal Tissue

To extend these studies to a more readily accessible source of respiratory tissue that might be routinely used in clinical studies and to validate these findings in a cohort of healthy adults, we used a noninvasive nasal sampling approach for assessing respiratory T-cell responses. None of the nasal sample donors had recent subclinical infection or prior history of infection with *B. pertussis* (Supplementary Table 1).

We recovered 20 000–50 000 CD3^+^ T cells by nasal swabbing (3 passes) of 20 healthy donors and a high proportion of these T cells were CD8^+^ and a smaller proportion were CD4^+^ (data not shown). A significantly greater proportion of the nasal tissue CD4 cells were T_RM_ cells when compared with tonsil (Figure 2*C*). Despite an interval of 20–30 years since pediatric vaccination, we were able to detect low numbers of T_RM_ cells that responded to *B. pertussis* antigens in nasal tissue of 80%–90% of donors. We found a significantly higher frequency of IFN-γ^+^ and IL-17^+^ CD4 T_RM_ cells in nasal mucosa of adults that received wP compared with aP vaccines as infants (Figure 2*D*).

These findings demonstrate the IFN-γ– and/or IL-17–producing *B. pertussis*-specific CD4 T_RM_ can persist in the respiratory tract for decades after pertussis vaccinations, and that this is most striking in adults that received the wP vaccine as children.

## DISCUSSION

This study provides the first evidence that CD4 T_RM_ cells are recruited and persist in the human respiratory tissues for 20–30 years after primary immunization with pertussis vaccines. Our findings also demonstrate that adults immunized with wP vaccines as infants have a significantly higher frequency of IFN-γ and IL-17–producing CD4 T_RM_ cells that respond to *B. pertussis* antigens in tonsil and nasal tissues than in individuals immunized with aP vaccines. In contrast, IL-13–producing T cells were dominant in the tonsil of individuals immunized with aP vaccines. This is consistent with studies on the blood, which demonstrated that wP vaccines generate Th1/Th17-polarized responses, whereas aP vaccine preferential prime Th2 cells [6, 7]. In our study population, who had not been recently boosted with pertussis vaccines, *B. pertussis*-specific CD4 T-cell responses were weak or undetectable in blood. Consistent with known persistence of T_RM_ cells in mucosal tissues, this suggests that memory CD4 T cells that respond to *B. pertussis* persist in respiratory tissue, but not in the circulation.

Epidemiological studies have shown that infants vaccinated with wP have more prolonged protection against disease than those immunized with aP vaccines [12]. Our demonstration that Th1/Th17 polarized responses persist in respiratory tissues following priming with wP vaccines is consistent with these findings. Studies in mouse models have shown that *B. pertussis* infection or immunization with wP vaccines promote accumulation of *B. pertussis*-specific CD4 T_RM_ cells in the lungs and nasal tissue and that these cells produce IFN-γ and IL-17 that mediate long-term protection against infection [4, 8]. Consistent with these findings, we observed that among the human T-cell repertoire in the respiratory tissue, which is exposed to a plethora of antigens, a small frequency of CD4 T_RM_ cells from wP-vaccinated individuals respond to *B. pertussis* antigens by producing IFN-γ or IL-17. This suggests that parenteral immunization of humans with a potent vaccine can drive T_EM_ cells to respiratory mucosal tissues and these cells persist as T_RM_ cells for decades after immunization.

This study is limited by a relatively small sample size. Furthermore, it is possible that the T_RM_ cells that we detected in human respiratory mucosal tissue were enhanced by subclinical infections with *B. pertussis*. Although we did not find any evidence of recent subclinical infection based on serum anti-PT IgG, subclinical infections may have occurred in the past. This may explain the IL-17 and IFN-γ–producing CD4 T cells responding to *B. pertussis* antigens detected in a small proportion of individuals that had been immunized as children with aP vaccines. However, this is unlikely to account for the presence of T_RM_ in a high proportion of wP-vaccinated donors. It is also possible that individuals may have been exposed to other *Bordetella* species and this could have activated cross-reactive T cells. Alternatively, T_RM_ cells may have been maintained in the respiratory tissue by indirect or bystander activation with unrelated respiratory pathogens. We have recently provided evidence of this in the mouse model [13].

Our findings, together with previous studies in animal models [4, 9], suggest that next-generation vaccines against pertussis should be designed to induce memory T cells as well as antibodies in the respiratory mucosa. Preclinical studies have shown that nasally delivered vaccines are more effective than parenterally delivered vaccine at inducing respiratory CD4 T_RM_ and preventing infection of the nasal mucosa [14, 15]. Because respiratory CD4 T_RM_ are central to protective immunity against *B. pertussis* infection [4, 9], assessment of cellular immunity in the respiratory tissues may be key to establishing correlates of protection. Noninvasive sampling of intraepithelial lymphocytes in the nasal tissue has the potential to be used to study vaccine or infection-induced mucosal T-cell immunity sequentially in clinical trials of new vaccines designed to prevent nasal colonization and asymptomatic transmission of *B. pertussis*.

## Supplementary Data

Supplementary materials are available at *The Journal of Infectious Diseases* online (http://jid.oxfordjournals.org/). Supplementary materials consist of data provided by the author that are published to benefit the reader. The posted materials are not copyedited. The contents of all supplementary data are the sole responsibility of the authors. Questions or messages regarding errors should be addressed to the author.

## Supplementary Material

jiae034_Supplementary_Data

## Notes

***Author contributions.*** K. H. G. M. conceived and supervised the study. R. M. L. cosupervised the research. S. H. provided clinical oversight and performed or supervised the procedures for the collection of tonsils. K. M. C. collected and processed samples and analyzed data. K. H. G. M. and K. M. C. interpreted the data and prepared the manuscript. All authors contributed to the critical review of the manuscript.

***Acknowledgment.*** We thank Barry Moran for assistance with flow cytometry.

***Financial support*.** This work was supported by a Wellcome-Health Research Board Irish Clinical Academic Training Programme Fellowship (to K. M. C.); Science Foundation Ireland Investigator Award (grant number 15/IA/3041 to R. M. M.); Wellcome Investigator Award (grant number 202846/Z/16/Z to R. M. M.); Science Foundation Ireland (grant numbers 16/IA/4468 and 12/RI/2340-7 to K. H. G. M.); and the PERISCOPE project, which has received funding from the Innovative Medicines Initiative 2 Joint undertaking (grant agreement number 115910). This Joint Undertaking receives support from the European Union Horizon 2020 Research and Innovation Programme, the European Federation of Pharmaceutical Industries and Associations, and Bill and Melinda Gates Foundation.
