# Supplementary material for: IL-17 and IFN-γ–producing Respiratory Tissue-Resident Memory CD4 T Cells Persist for Decades in Adults Immunized as Children With Whole-Cell Pertussis Vaccines
Source: J Infect Dis. 2024 Jan 30;230(3):e518–23. doi: 10.1093/infdis/jiae034 (PMC11420794; doi:10.1093/infdis/jiae034)
Supplement: jiae034_Supplementary_Data [file jiae034_supplementary_data.pdf]

IL-17 and IFN-γ-producing respiratory tissue resident memory CD4 T cells persist for decades in adults immunized as children with whole cell pertussis vaccines

Karen N McCarthy, Stephen Hone, Rachel M. McLoughlin and Kingston HG Mills

Supplementary Data

Supplementary Table 1: Demographic characteristics of human donors

Baseline characteristic	wP cohort (n= 10)	aP cohort (n=10)	p-value
**<u>Tonsil cohort</u>**			
Gender			
Female	n=6 (60%)	n=7 (70%)	0.6
Male	n=4 (40%)	n=3 (30%)	
Age (in years)			
Median (IQR)	30 (27-34)	21 (19-24)	<0.001
**Indication for Tonsillectomy**			
Recurrent tonsillitis	10 (100%)	10 (100%)	-
Obstructive sleep apnoea / Tonsillar hypertropthy	0	0	-
**Sample characteristics**			
Tonsil sample weight (g)	7.5 (4.7)	5.8 (5.9)	0.5
Tonsil mononuclear cell yield per gram of tissue (mean, SD)	33.2 x 10^6 (57.2)	33.2 x 10^6 (47.0)	0.9
Anti-Pertussis toxin Serum IgG (Median, IQR)	11.68 (5-30)	16.23 (9-29)	0.6
**<u>Nasal cohort</u>**			
Gender			
Female	n=8 (80%)	n=9 (90%)	0.55
Male	n=2 (20%)	n=1 (10%)	
Age (in years)			
Median (IQR)	32 (30-32)	25.5 (22.7-26.3)	<0.001
Sample characteristics			
Nasal CD3+ T cell yield per stimulation condition	13941 (8374-17474)	17225 (8318-21825)	0.1
PBMC CD3-depletion purity mean% (SD)	96% (1.72)	95.5% (3.2)	0.37
Anti-Pertussis toxin Serum IgG (Median, IQR)	5 (5-9)	5 (5-5)	0.5

Supplementary Table 2: Exclusion criteria

Recruitment data	
**Tonsil cohort**	
Total number of patients approached	35
Declined to participate	3
Excluded	0
Primary or Secondary immunodeficiency	0
Malignancy	1
Tonsil hypertrophy	2
Receipt of antibiotics <30 days	1
Prior history of *B. pertussis* infection	0
Evidence of current *B. pertussis* infection *(B. pertussis PCR positive on nasopharyngeal swab)*	0
B. pertussis booster <12 months	3
Any vaccination <1 month	5
Incomplete/Unknown childhood vaccination schedule	0
Exclusion criteria included underlying primary or secondary immunodeficiency, malignancy, receipt of antibiotics within 30 days of procedure, tonsil hypertrophy as indication for tonsillectomy, prior history of *B. pertussis* infection, evidence of current *B. pertussis* infection via PCR on nasopharyngeal swab at the time of procedure, booster pertussis vaccination in past 12 months or any vaccination in the past 1 month. Participants with an incomplete or unknown childhood immunisation schedule were also excluded. Data were collected from the clinical notes and from the patient including date of birth, gender, procedure and indication, country and year of primary childhood immunisation, additional immunisations or exclusions and current respiratory symptoms were recorded. Where possible patients or parents/guardians were asked to provide vaccination records to confirm vaccination status. Where the original vaccination record was not available, information was collected from the patient including date, location and number of vaccinations.	

Supplementary Table 3: Antibodies and other reagents

Antibody	Fluorochrome	Clone	Supplier	Catalogue Number
**Flow cytometry antibodies**				
Anti-human CD103	FITC	Ber-ACT8	Biolegend	350203
Anti-human CD4	PerCP e710	SK3	eBiosciences	4320262
Anti-Human IL-17A	APC	eBio17B7	eBiosciences	B205752
Anti-human CD3	APC-H7	SK7	eBiosciences	7158521
Anti-human CD154	BV421	24-31	BioLegend	310824
Anti-human IL-13	BV421	JES10-5A2	BioLegend	501916
Anti-human TCR $\gamma\delta$	BV421	B1	BioLegend	331218
Live/Dead Fixable Aqua	BV510	NA	Invitrogen	2068285
Anti-human CD8	BV605	SK1	BioLegend	344741
Anti-human CD45RO	BV650	UCHL1	BioLegend	B239721
Anti-human CCR7	PE	G643H7	BioLegend	B243036
Anti-human IFN-γ	PE-Cy7	4S.B3	eBiosciences	6145884
**Cytokine secretion assays**				
Anti-human IFN-γ	FITC	-	Miltenyi Biotech	130-090-433
Anti-human IL-17	APC	-	Miltenyi Biotech	130-094-536
**Antigens**				

Antigen	Concentration *in vitro*	Supplier
Filamentous Hemagglutinin	1 µg/ml	The Native Antigen Company
Staphylococcal Enterotoxin B	1 µg/mL	Sigma Aldrich
Sonicated *B. pertussis*	10 µg/mL	Produced in-house by heat killing and sonicating *B. pertussis* (BP338)

**Other Reagents**		
**Reagent**	**Concentration *in vitro***	**Supplier**
Anti-human CD28	1 µg/ml	eBioscience
Anti-human CD49d	1 µg/ml	eBioscience
Brefeldin A	5 µg/ml	Sigma Aldrich
Monensin	5 µg/ml	BD Golgi Stop
FoxP3 Staining Kit	NA	eBioscience
Live/Dead Aqua	1:600 (PBS)	ThermoFisher
FACS Compensation Beads	1:10	BD
ArC Amine Reactive Beads	NA	ThermoFisher
Human TruStain FcBlock	1:20	BioLegend
Arc Amine Reactive Compensation Bead Kit	NA	Invitrogen
Cytostim	20 µl/ml	Miltenyi-Biotech

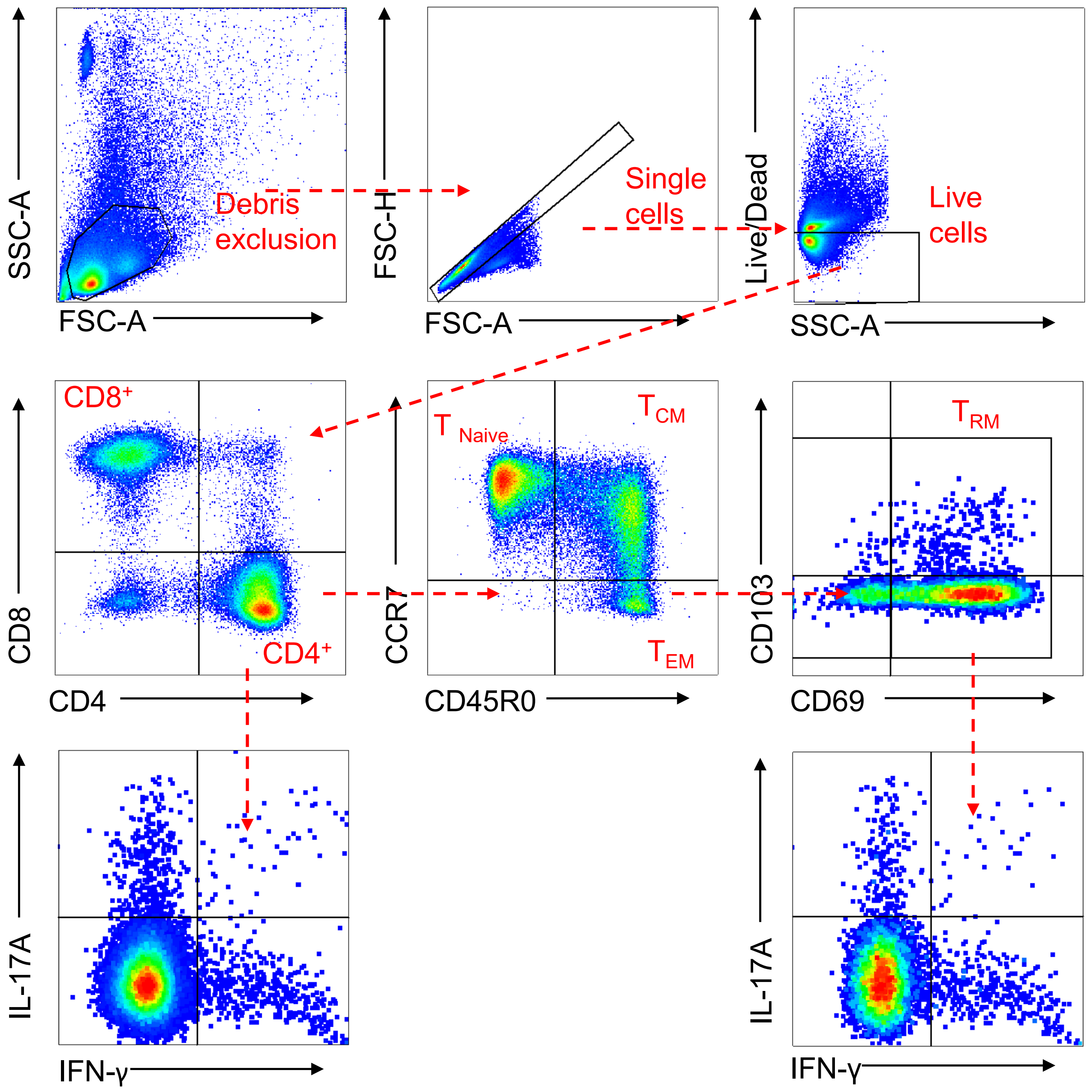

Supplementary Figure S1. Gating strategy for quantifying cytokine producing tonsil CD4 T cells and CD4 T_{RM} cells.

Lymphocytes were defined by forward scatter (FSC) and side scatter (SSC) density plots based on size and granularity. Doublets were excluded based on SSC-A vs SS-H density plots. Dead cells were excluded based on Live/Dead Fixable Aqua staining. $CD4^{+}$ T cells were identified by positive staining for CD4. Effector memory T (T_{EM}) cells were identified based on positive staining for CD45RO and $CCR7^{low}$. T_{RM} cells were defined based on expression of CD69 with or without co-expression of CD103. Cytokine producing cells were identified via intracellular cytokine staining. Fluorescence minus one (FMO) controls were used to define positive and negative populations.

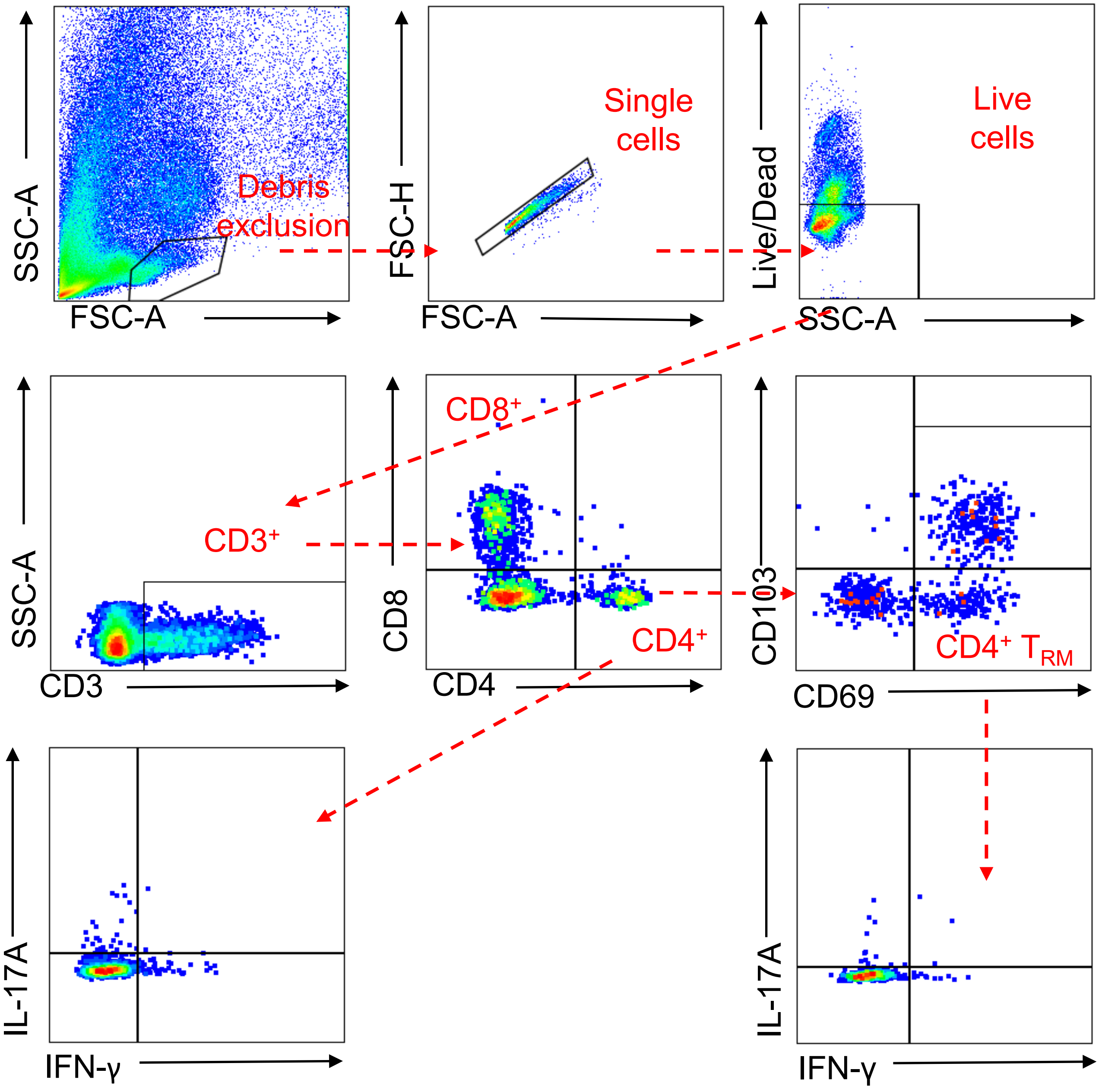

Supplementary Figure S2. Gating strategy for quantifying cytokine producing CD4 T cells and CD4 T_{RM} cells in nasal tissue samples.

Lymphocytes were defined by forward scatter (FSC) and side scatter (SSC) density plots based on size and granularity. Doublets were excluded based on SSC-A vs SS-H density plots. Dead cells were excluded based on Live/Dead Fixable Aqua staining. $CD3^{+}$ T cells were identified based on positive staining for CD3 . $CD4^{+}$ cells identified by positive staining CD4 and negative staining for CD8. T_{RM} cells were defined based on expression of CD69 with or without co-expression of CD103. Cytokine producing cells were identified via cytokine secretion assay. Fluorescence minus one (FMO) controls were used to define positive and negative populations.
